# Functional outcome of the anterior vaginal wall in a pelvic surgery injury rat model after treatment with stem cell-derived progenitors of smooth muscle cells

**DOI:** 10.21203/rs.3.rs-4172308/v1

**Published:** 2024-06-11

**Authors:** Yiting Wang, Yan Wen, Kayla Kim, Hugo Wu, Jerry Zhang, Amy D. Dobberfuhl, Bertha Chen

**Affiliations:** Stanford University School of Medicine; Stanford University School of Medicine; Stanford University School of Medicine; Stanford University School of Medicine; Stanford University School of Medicine; Stanford University School of Medicine; Stanford University School of Medicine

**Keywords:** progenitors of smooth muscle cell (pSMC), induced pluripotent stem cell(iPSC), organ bath myography, recurrent vaginal prolapse, pelvic organ prolapse

## Abstract

**Background:**

Stem-cell-derived therapy is a promising option for tissue regeneration. Human iPSC-derived progenitors of smooth muscle cells (pSMCs) have limited proliferation and differentiation, which may minimize the risk of *in vivo* tumor formation while restoring smooth muscle cell deficiencies. Up to 30 % of women who suffer from recurrence of vaginal prolapse after prolapse surgery are faced with reoperation. Therefore, there is an unmet need for therapies that can restore vaginal tissue function. We hypothesize that human pSMCs can restore vaginal function in a vaginal-injury rat model.

**Methods:**

Female immune-compromised RNU rats were divided into 5 groups: intact controls (n=12), VSHAM (surgery + saline injection, n=33), and cell-injection group (surgery + cell injection using three patient pSMCs lines, n=14/cell line). The surgery, similar to what is done in vaginal prolapse surgery, involved ovariectomy, urethrolysis, and vagina injury. The vagina, urethra, bladder dome and trigone were harvested 10 weeks after surgery (5 weeks after injection). Organ bath myography was performed to evaluate the contractile function of vagina, and smooth muscle thickness was examined by tissue immunohistochemistry. Collagen I, collagen III, and elastin mRNA and protein expressions in tissues were assessed.

**Results:**

When compared to the VSHAM group, cell-injection groups showed significantly increased vaginal smooth muscle contractions induced by carbachol (groups A and C) and by KCl (group C), and significantly higher collagen I protein expression in the vagina (groups A and B). Elastin mRNA and protein expressions in the vagina did not correlate with injection group. In the urethra, mRNA expressions of collagen I, collagen III, and elastin were all significantly higher in the cell-injection groups compared to the VSHAM group. Collagen I protein expression of the urethra was also higher in the cell-injection group compared to the VSHAM group. Elastin protein expression in the urethra did not correlate with injection group.

**Conclusions:**

Human iPSC-derived pSMCs improved contractile function of the post-surgery vagina. Additionally, pSMC injection modulated collagen I, collagen III and elastin mRNA and protein expressions in the vagina and urethra. These findings suggest that pSMCs may be a possible therapy for vaginal prolapse recurrence after surgical intervention.

## Background

Pelvic organ prolapse (POP) is a debilitating condition characterized by the downward movement of the vaginal and/or uterus through the vaginal opening. Other pelvic structures such as the bladder, bowel, and rectum can also descend behind the vagina and uterus resulting in a variety of genito-urinary and bowel symptoms that severely impact quality of life. POP incidence is 40% in women between ages 45 to 85, and increases with advancing age[1].

The muscles and connective tissues in the pelvic floor support the pelvic organs, maintaining their position and function. The levator ani muscle is the main support of the pelvic floor. The vagina also plays an important role in supporting pelvic organs, with its smooth muscle fibers connected to surrounding smooth muscles and attached to the outer and inner surfaces of the levator ani[2]. Factors such as vaginal childbirth, chronic increased abdominal pressure, pelvic surgery, and aging can weaken the support of the pelvic floor through tissue injury/atrophy and cell apoptosis[3]. Smooth muscle has also been implicated in the pathophysiology of POP[4]. Histologically, the vaginal wall of women affected with POP is deficient in elastin fibers and smooth muscle cells, resulting in stiffer biomechanical properties compared to the vaginal wall from controls.

POP treatment options rely mostly on surgical attempts using patient’s own tissues or synthetic meshes to reinforce or support the deficient pelvic structures and vaginal wall. POP is associated with an 11% lifetime risk of surgical treatment. Despite surgical intervention, 29% of treated women will require reoperation for recurrence of prolapse[5, 6]. The anterior vaginal wall is a common site of prolapse recurrence after POP surgery [7]. Currently, there are no treatments to prevent recurrence after surgery.

Given the limited options for the treatment of recurrent prolapse and their suboptimal results, there is great interest in using stem cells to restore deficient pelvic tissues. Different types of cells, such as mesenchymal stem cells (MSCs), induced pluripotent stem cells (iPSCs), and stem cell-derived progenitor or precursor target cells have been considered. The advantages of iPSC-based therapies include: 1. Cells can be autologous, devoid of ethical and immunological concerns, 2. Cells can be expanded in large numbers for therapeutic dose, and 3. Homogeneous cell populations can be produced with defined protocols. However, iPSCs have the potential to develop into teratomas *in vivo*. To minimize this risk, many groups have differentiated these cells into progenitors of specific cell types for clinical translation, such as iPSCs-derived progenitors of smooth muscle cells (pSMCs). Human iPSC-derived pSMCs transplanted into the urethra of a stress urinary incontinence (SUI) rat model restored urethral sphincter function by regenerating smooth muscle cells and inducing native tissue elastin/collagen III remodeling [8]. Given these encouraging results, we hypothesize that pSMCs may also be effective in restoring function to the surgically injured vagina.

Therefore, in this study, we sought to test whether iPSC-derived pSMCs injected into the vagina of a surgically-injured-vagina rat model might improve the contractile function of the vagina and modulate extracellular matrix (ECM) proteins, such as elastin and collagen, in the vagina.

## Materials And Methods

### 1. Reprogramming of iPSCs and differentiation of pSMCs

Skin biopsies were collected from three female patients (ages 40–70). Dermal fibroblasts were reprogrammed to iPSCs using a modified mRNA/miRNA reprogramming method. mRNA encoding *Oct4, Klf4, Sox2, c-Myc*, and *Lin28* as well as miR-302/367 cluster were transduced [9].

The iPSCs at passage 2 (P2) were differentiated into pSMCs using the feeder-free vascular endothelial progenitor protocol previously described[10]. pSMCs were cultured on human placenta-derived collagen IV (Sigma-Aldrich, Missouri) and expanded in DMEM/F12 HEPES with 5% fetal bovine serum (FBS). pSMCs at P4 were used for injection.

### 2. Generation of the vaginal-injury rat model and cell injection into the vagina

Healthy female immunodeficient Rowett Nude rats (RNU, Charles River Laboratories, Hollister, CA) weighing 150–200g were used to create the vaginal-injury model as described in our published study [11]. Animals were maintained at the Stanford University Research Animal Facility in accordance with Stanford University’s Institutional Animal Care and Use Committee guidelines. The animals were anesthetized with the intraperitoneal injection of ketamine (30–100mg/kg) and xylazine for the surgery or with inhalation of 1–4% isoflurane for the cell injections.

The animals were euthanized with inhalation of carbon dioxide.

Briefly, an abdominal midline incision was made. Adnexal vascular supply was ligated and ovaries resected. To expose the anterior vagina, the urethra was circumferentially detached from the anterior vaginal wall and pubic bone by sharp dissection. To mimic the vaginal injury caused by prolapse surgeries, two cuts were made in the anterior vaginal muscular layer without penetrating into the vaginal lumen. This area was then clamped with a hemostat for 5 minutes. The abdominal incision was then closed. The rats were checked daily for a week after surgery, and then once a week until euthanasia. Rat cages were housed in the same room and shelf to minimize confounders.

Cell injection was performed at 5 weeks post-surgery to allow for inflammatory changes to subside. In the VSHAM group, we injected saline into both sides of anterior vagina wall. In the cell-injection groups, 2 million cells in 200ul sterile saline were injected (1 million/100ul /each side). Unfortunately, the injection procedure could not be performed in a randomized fashion because the cells for each group needed to be prepared in one batch as cell survival is time sensitive.

The rats were assigned sequentially into five experimental groups as they arrived from Charles River Laboratories. Randomization was not feasible because of the sporadic availability and supply of the RNU rats during the Pandemic. The five experimental groups were: (1) intact controls (control group, n=12). (2) vaginal-injury model injected with saline (VSHAM group, n=33). (3–5) vaginal-injury model injected with pSMCs derived from patient A, B, and C (cell-injection groups A, B, and C, n=14/ group). Patients A and C are in their 40’s and patient B is in her 70’s of age. Rats were monitored for 5 weeks after injection.

#### Sample size calculation:

our primary outcome measure was the vaginal tissue contractile response to KCl stimulation at 40mM. Based on our preliminary data, we estimated the expected effect size to be 0.2 g/cm^2^ (one times the contraction size of VSHAM) and the standard deviation to be 0.2. This gives us a standard effect size of 1.00 which yields a sample size of 12 rats per group for power of 80% and two-tailed a of 0.10[12]. The higher number of rats in VSHAM group is due to the need for comparisons with sham rats for the multiple assays.

### 3. In vivo bioluminescence imaging (BLI) of transplanted pSMCs

A human luciferase-tagged iPSC line (Huf-5) was used to differentiate pSMCs. These luciferase-tagged pSMCs were injected bilaterally into the anterior vagina (1 million cells/100uL sterile saline/site) of control (n=4) and vaginal-injury rats (n=4) 5 weeks after surgery to track the human cells. D-luciferin (Biosynth, Itasca, IL) was injected intraperitoneally 375 mg/kg body weight before image acquisition on Day 0, Day 2, Week 1, Week 2, and Week 5. Transplanted cell survival *in vivo* was monitored via bioluminescence imaging with Lago X (Spectral Instruments Imaging, Tucson, AZ). The photons emitted from luciferase-expressing cells were collected with integration times of 2 minutes.

### 4. Detection of human gene sequence in harvested tissues from the BLI study using Alu-sequence-PCR Assay

The bladder, urethra, vagina, and uterus were harvested from the rats used for BLI cell tracking after euthanasia on Day 2, Week 1, Week 2, and Week 5. Genomic DNA was extracted from tissue homogenates following the manufacturer’s instruction (QIAGEN, Redwood, CA). The amount of human DNA in each sample was quantified by amplification of a human-specific DNA sequence *Alu* (Primer sequences are shown in Table 1). Brilliant SYBR Green PCR method was used to perform PCR using AriaMx (Agilent Technologies, Santa Clara, CA). All PCR reactions were performed in triplicate for fifty cycles. pSMC numbers per 25ng of DNA was calculated using a calibration curve with defined numbers of pSMCs.

### 5. Tissue collection and organ bath myography of the five experimental groups

Vagina, bladder dome, trigone and urethra were harvested 10 weeks post-surgery (5 weeks post-injection). Below is a description of the tissue section used for the different assays.

#### (1) Vagina

- Proximal portion: one half was fixed in paraffin and used for H&E, elastin, and h-Caldesmon staining, and the other half was used for RT-qPCR.
- Middle portion: used for organ bath myography first, followed by ELISA.
- Distal portion: stored in −80°C freezer.

#### (2) Bladder Dome and Trigone

- Bladder dome and trigone were evaluated with organ bath myography first, followed by histology, RT-qPCR and ELISA.

#### (3) Urethra

- Proximal portion: fixed by OCT (optimal cutting temperature) compound and used for elastin staining.
- Middle portion: RT-qPCR
- Distal portion: ELISA

The organ bath myography methodology was described in detail in our previous publication[11]. Briefly, the middle portion of the vagina was mounted circumferentially, and the bladder dome and trigone were mounted longitudinally. The contractile responses were monitored using a custom-made isometric force transducer, and signals were recorded using Lab Chart 7 (AD Instrument, Colorado Springs, Colorado). Firstly, we assessed tissue contraction using potassium chloride (KCl) solution: 40mM KCl for vagina and 160mM KCl for bladder tissues. After the tissues reverted to their resting tension by washing with Krebs buffer, we assessed the contraction by using carbachol (Sigma-Aldrich, Missouri), a nonselective muscarinic receptor agonist, in increasing concentrations of 0.625μM, 1.25μM, 2.5μM, 5μM, 10μM, 20μM. Strips were washed again before verifying true carbachol-stimulated response by adding 1μM atropine, and carbachol (20uM) 5 minutes post atropine. Tissues were washed and viability checked again using initial KCl concentrations. Tissue contractility data were normalized to tissue area, expressed as tension per unit of tissue area (g/cm^2^).

### 6. Reverse-Transcription Quantitative Polymerase Chain Reaction (RT-qPCR)

RNA extraction and RT-qPCR were performed as in our published manuscript[11]. RT-qPCR was used to evaluate mRNA expression of elastin, collagen I, and collagen III. Primer sequences are shown in Table 1. RT-qPCR was performed in duplicates on Aria Mx Real-Time PCR, using Brilliant SYBR Green PCR Master Mix as described previously[8]. GAPDH was used as an endogenous reference.

### 7. Enzyme-linked immunosorbent assay (ELISA)

Protein expression of elastin, collagen I, and III was quantified in duplicate with ELISA kits (Lifespan Biosciences, Seattle, WA) as per manufacturer’s instructions. Optical absorbance was measured with a spectrophotometer, SpectraMax M3 (Molecular Devices, San Jose, CA). Quantification of target proteins was calculated based on its standard curve, then normalized to the concentrations of the protein (mg/ml) in the samples.

### 8. Elastin staining and qualitative scoring of elastin in vaginal and urethral tissues

Proximal vaginal tissues were embedded in paraffin and proximal urethral tissues were embedded in OCT. Elastin staining on paraffin and OCT were performed as described previously [13, 14]. Briefly, elastin fibers were stained in Weigert’s Resorcin-Fuchsin solution (Electron Microscopy Sciences, Hatfield, PA). Cell nuclei were stained with Weigert’s iron hematoxylin working solution (Poly Scientific R&D Corporation, Bay Shore, NY). After washing in running water, the slides were placed in van Gieson’s solution for 3–5 min for collagen fiber staining.

The slides were scored by four people for elastin fiber length (1= short, 2 =moderate, 3 = long), thickness (1 = thin, 2 = moderate, 3 = thick), and density (1 = sparse, 2 = moderate density, 3 = dense). All four examiners were blinded to the group designation.

### 9. h-Caldesmon (h-CALD) staining and vaginal smooth muscle layer quantification

We performed h-CALD, a marker for SMC’s, immunohistochemical staining to evaluate SMC. The slides of proximal vaginal tissue were deparaffinized, retrieved and blocked. The mouse anti-h-CALD antibody (1:100, Santa Cruz Biotechnology, Santa Cruz, CA) was incubated at 4°C overnight, and then incubated with the second antibody (horse anti-mouse-biotin,1:50 Themo Fisher Scientific, Waltham, MA). The ABC kit was used to develop the red color (Vector Laboratories, Burlingame, CA).

Because of the surgical procedures, some parts of the vagina were much thinner than other parts, so we measured the thinner and thicker parts separately. The thinner part of the vagina is likely to be the surgically injured area. Six measurements were taken on the thinner part and another 6 taken on the thicker part. Zen software (blue edition, version 3.4, ZEISS, White Plains, NY) was used to measure the thickness. This was performed by two separate people. Thus, the average of 12 measurements (from the examiners) was reported for each part of the vagina (thinner and thicker).

### 10. Data Analysis

Statistical analysis was performed using JMP software version 17 (SAS Institute, Inc., Cary, NC) The results are expressed as the mean±SD. The nonparametric Wilcoxon test was applied for statistical comparisons between groups. All data points were included in the analyses. Statistical significance was set at p<0.05.

The work has been reported in line with the ARRIVE guidelines 2.0

## Results

### 1. Short-term human cell tracking and Alu-seq-PCR after vaginal injection in the control rat and vaginal-injury rat model.

**Figure 1:**
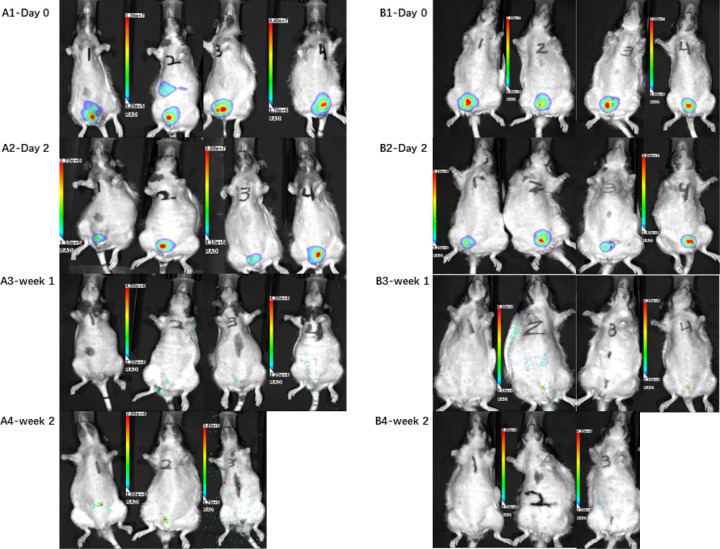
In vivo bioluminescence imaging of transplanted Huf5-luc pSMCs. A is the control group (n=4), B is the vaginal-injury group (n=4)., the signals were detected in all the rats in the pelvic on Day 0 and 2 (A1–2 and B1–2). By Week 1, weak signals were detected in some of the rats. Faint signals were only detected in the control group by Week 2 (A4 and B4).

BLI signal was observed in the pelvis in the area of the bilateral vaginal injection. The signal was faintly present for 1 week in the vaginal-injury group and 2 weeks in the control group (Figure 1). While there was no BLI signal, *Alu*-seq-PCR showed a calculated total of 900 human cells in the urethra by the end of 5 weeks in the control group. In the vaginal-injury group, no human cells were detected by BLI or *Alu*-seq-PCR after week 1.

### 2. Functional effect of vaginal pSMCs injection on the smooth muscle of the vagina, bladder dome, and trigone

Several rats died due to surgical complications, so the numbers in each group used for analysis were as follows: 33 in VSHAM group, 11 in cell-injection group A, 12 in cell-injection group B, 12 in cell-injection group C.

#### Vagina

**Figure 2:**
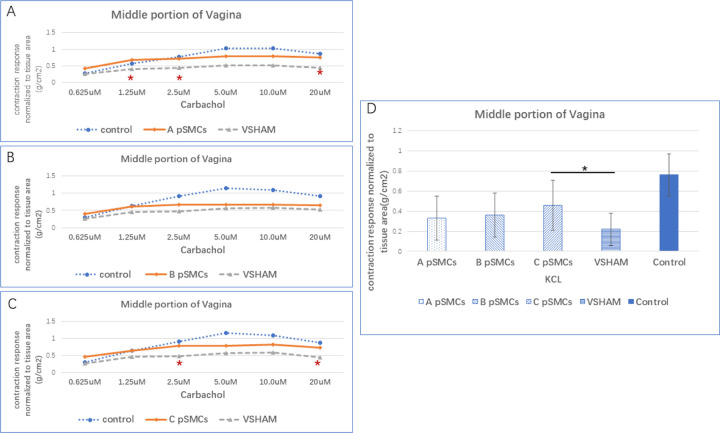
Organ Bath Myography of vagina smooth muscle. 2.A-C vagina middle contraction response induced by different concentrations of carbachol, normalized to tissue area, in different pSMCs groups (Patient A, B and C). 2.D contraction response induced by KCl in different groups. * significant difference compared to VSHAM group, P=0.05.

Two of three cell-injection groups (A and C) showed that the contraction response to carbachol stimulation, normalized to the area of the vagina, was significantly stronger than the VSHAM group at 2.5μM and 20μM carbachol concentration (P = 0.040). In group A, it was also significantly stronger at the 1.25μM concentration P=0.0448)(Figure 2A–2C, Table S1). The KCl-induced contraction was significantly stronger in the cell-injection group C (P=0.0446) compared to the VSHAM group, while cell-injection A and B groups showed a higher trend compared to the VSHAM group (Figure 2D, Table S1).

**Figure 3:**
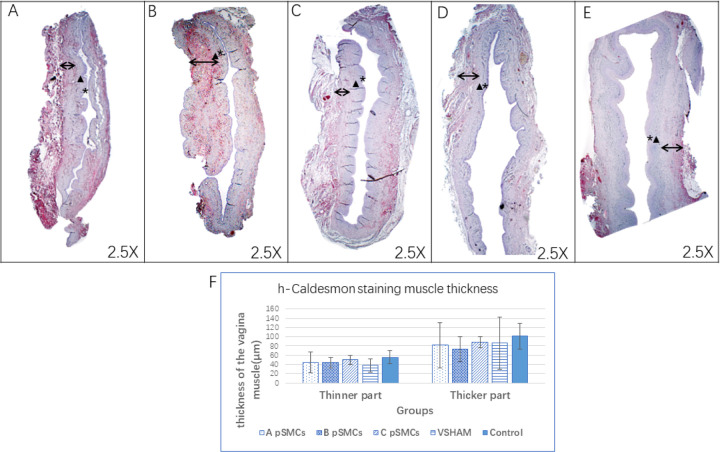
h-Caldesmon staining of the vagina. 3.A-C: cell-injection group A,B and C. 3.D: VSHAM group. 3.E: control group. *: Mucosal layer. ▲: Laminate Propria layer. ↔: Muscular Layer. 3.F The mean smooth muscle thickness both at thinner part and thicker part of the vagina.

Based on immunohistochemical staining, the thickness of the thinner part of the vagina in all 3 cell-injection groups tended to be thicker than that of the VSHAM group, although not significant (Figure 3. VSHAM: 38.0±14.10μm, cell-injection group A: 44.4±22.36μm, P=0.575. group B: 43.9±11.48μm, P=0.379. group C: 49.88±9.68μm, P=0.065).

#### Bladder dome and trigone

**Figure 4:**
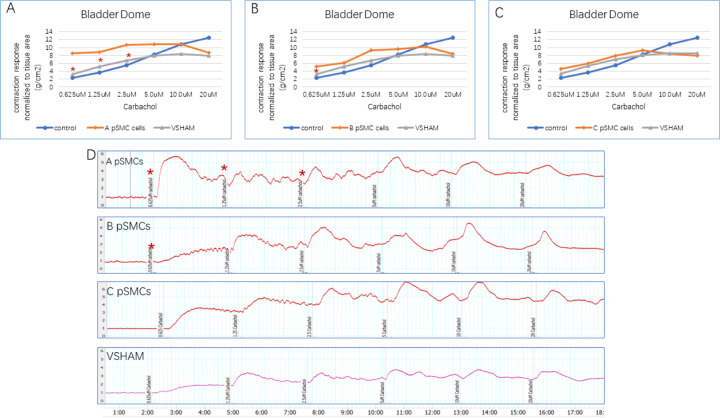
Organ Bath Myography of bladder dome. 4.A-C bladder dome contraction response induced by different concentrations of carbachol, normalized to tissue area, in different pSMCs groups (cell-injection group A, B and C). * P=0.05. 4.D contraction with different concentrations of carbachol stimulation.

Carbachol-induced contraction response normalized to the area of the bladder dome in cell-injection groups was significantly stronger compared to the VSHAM group at low concentrations (0.625μM in groups A and B, 1.25μM and 2.5μM in group A, P=0.037) (Figure 4, A-D) There was no significant difference in bladder trigone between the VSHAM group and any of the cell-injection group.

### 3. Effect of pSMCs injection on gene and protein expression of collagen I, collagen III, and elastin in the vagina.

Compared to VSHAM vaginas, collagen I, III, and col I/III mRNA expression in cell-injection groups were not consistently increased or decreased (Table 2).

However, the protein expression of collagen I was consistently increased in the vaginas of cell-injection groups compared to VSHAM group with group A and B being significantly elevated (P=0.029). Collagen III and col I/III protein expression were not consistent.

Neither mRNA nor protein expression of elastin in the three cell-injection groups showed a consistent trend when compared to the VSHAM group (Table 2). Elastin staining score for both the amount and length of elastin fiber also did not show a difference between groups.

### 4. Effect of pSMCs injection on gene and protein expression of collagen I, collagen III and elastin in the urethra.

mRNA expression of collagen I and III in the three cell-injection groups were significantly higher than the VSHAM group (P=0.001, Table 3). The col I/III mRNA expression ratio was also significantly lower for all three cell-injection groups compared to the VSHAM group (P<0.0036, Table 3), indicating that collagen III expression was higher than that of collagen I.

Protein expression of collagen I in one out of three groups was significantly higher than VSHAM group (P=0.0477) with the remaining two cell-injection groups showing increasing trends. However, the protein expression of collagen III and col I/III ratio was not consistent for the three cell-injection groups (Table 3).

pSMCs promoted significantly increased elastin gene expression in all three cell-injection groups compared to the VSHAM group (p=0.001). No significant trend was seen in elastin protein expression in all three cell injection groups (Table 3).

**Figure 5:**
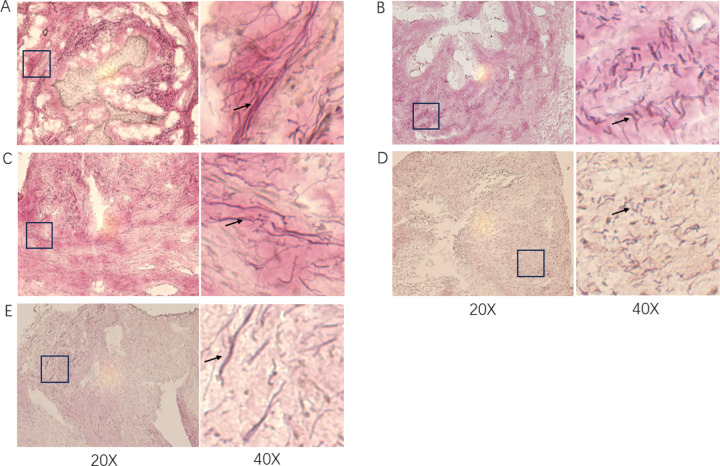
Elastin staining of the urethra. 5.A-C: cell-injection groups A, B and C. 5.D: VSHAM group. 5.E: Control group. The arrows indicate the black elastin fiber.

Qualitative analysis of elastin in the urethra (Figure 5), revealed that the elastin fiber length score in cell-injection group B was significantly higher than VSHAM group (P=0.0074), but the other two cell injection groups were similar to the VSHAM group. The amount and thickness scores were similar for all groups.

### 5. Effect of pSMCs on gene and protein expression of, collagen I, collagen III, and elastin in the bladder

The mRNA expression of collagen I, collagen III, and col I/III ratio in the bladder dome did not show a consistent trend in the three cell-injection groups compared to the VSHAM group. Gene expression of elastin in the bladder dome was significantly lower in one out of three cell-injection groups compared to the VSHAM group (P=0.0005) (Table S2).

mRNA expressions of collagen I (group C, p=0.0035) and III (group B and C, p,0.0134) in the bladder trigone were increased significantly with the remaining groups showing an increased trend compared to the VSHAM group. Col I/III ratio were all decreased, with group B showing significance (p= 0.0404), compared to VSHAM group. However, protein expressions did not show a consistent trend.

No significant differences were observed for elastin mRNA expression of the bladder trigone in the cell-injected groups compared to the VSHAM group. Elastin protein expression in the bladder trigone was significantly lower in two out of three cell-injection groups (group A and C) compared to the VSHAM group (P=0.013) (Table S2).

## Discussion

Smooth muscles play an important role in the function of the vaginal wall [15]. The content of smooth muscle cells (SMCs) in the vagina decreases with aging and is less in women with pelvic organ prolapse (POP) compared to un-affected women[16–18]. This decrease in smooth muscle content, organization, and composition can negatively affect vaginal tone and contractility, which are characteristic of prolapsed vaginal tissue [15, 18]. This may be due to the effect of aging on the regenerative ability of muscle by reducing both stem cell pool and functionality [19]. Smooth muscle cells and fibroblasts also contribute to the extracellular matrix of pelvic tissues. Others have also documented differential expression of extracellular matrix proteins in pelvic tissues of women with and without prolapse [20]. Together, these data suggest that several mechanisms contribute to the presence of pelvic prolapse.

Cell therapy, which aims to improve the proliferation or content of SMCs, may be an approach to address SMC loss or decline. There are limited studies using muscle cells to address deficiencies in SMCs. Skeletal muscle-derived cells (SMDCs) injected into the external anal sphincter improved sphincter-related fecal incontinence due to external anal sphincter damage and/or atrophy in both men and women[21]. While bone marrow mesenchymal stem cell-derived smooth muscle cells cultured with rat aortic SMCs in a 3D collagenous milieu showed significant pro-elastogenic and anti-proteolytic effects and higher contractility, as well as a greater amount of elastin production [22]. These publications suggest that SMC-based therapy not only can address cell deficiency but may modulate the abnormal ECM observed in prolapsed pelvic tissues.

The focus of our group is translation of human iPSC technology for pelvic floor disorders. Our previous work demonstrated that periurethral injection of human iPSC-derived progenitors of smooth muscle cells (pSMCs) can facilitate the restoration of sphincter SMCs and function in a stress urinary incontinence (SUI) rat model [8]. Given that the anterior vagina is the most common site for prolapse recurrence after pelvic organ surgery, in this study, we hypothesized that iPSC-derived pSMCs may have a similar regenerative effect in the surgically injured anterior vagina. Organ bath myograph with carbachol and KCl stimulation was used to evaluate the function of the vagina. Carbachol is a cholinergic agonist that mimics the action of the neurotransmitter acetylcholine which induces smooth muscle contraction, and KCl induces smooth muscle contractile function by direct membrane depolarization [23, 24]. Our data show that both carbachol and KCl induced stronger contractile responses in the cell-injection groups compared to the saline-treated group.

We also examined the thickness of smooth muscle in the vagina with immunohistochemistry staining for a SMC marker (h-CALD). We observed that vaginal thickness was slightly higher in the cell-injection group compared to the VSHAM group in the thinner part of the vagina, although this difference was not statistically significant. The lack of significance may be due to the high variability of the measurements and the qualitative nature of this assessment modality. Future studies would benefit from a more quantitative method of assessing SMC change.

Although we did not directly damage the bladder, we separated the bladder and urethra from the uterus and anterior vagina during model creation. Therefore, we also evaluated the bladder in this study. We found that bladder dome contractions in response to carbachol were stronger in most of the cell-injection groups at low concentrations of carbachol and KCl compared to the VSHAM group. However, there was no difference in the bladder trigone between the cell-injection and VSHAM groups. The bladder trigone has two muscle layers, an outer layer of detrusor muscle fibers that is parasympathetically innervated and an inner smooth muscle layer that receives noradrenergic sympathetic innervation [25]. We were not able to evaluate nonadrenergic sympathetic innervation due to the length of time that the tissues were exposed to organ bath with the carbachol and KCl stimulation, therefore we cannot comment on whether there might be changes in sympathetic innervation of the bladder trigone.

The extracellular matrix (ECM) provides the substrate on which cells migrate, proliferate, and differentiate [16]. This substrate is dynamic and undergoes constant remodeling in response to external forces, therefore, it also modulates the biomechanical properties of the tissue. Collagen I and III are two major protein subtypes in pelvic tissues. Collagen I confers tensile strength, while collagen III confers flexibility and distensibility. Both are present in tissues that are subjected to periodic stress[10, 26].

The ECM in the vagina changes with age, trauma, and conditions like POP. The literature supports differential expression of ECM components in women with and without POP, however, the data are not consistent. Some studies document increase in fibroblasts, collagen, and elastin fibers with aging, while others have observed poor-quality collagen or elastin fibers in women with POP[16, 18, 27]. Moalli et al observed an increase in collagen III in the sub-epithelium and muscular layer of the vaginal wall in patients with POP[20]. Chi’s study demonstrated that a severe prolapse is associated with imbalanced collagen synthesis, degradation and deposition. Protein content of both collagen I and collagen III in the vagina tissue was reduced, [28]. Söderberg investigated the overall collagen content in paraurethral ligaments and found it to be lower in patients with prolapse in comparison to those without[29].

In our study, collagen I protein expression in the vagina significantly increased in the cell-injection groups compared to the saline-injection group, but collagen III and col I/III ratio expression was variable. This suggests an increase in vaginal wall tensile strength in the cell-injected groups.

Elastic fibers are made up of multiple components, including elastin, fibrillins, and fibulins. These fibers also contribute to the integrity of the vaginal wall by providing extensibility and the ability to recoil with stretch. Hence, intact and mature elastic fibers may also be critical for maintaining smooth muscle cell contractility and vaginal biomechanical integrity[23]. There is documentation of decreased elastic fiber components, elastin, and fbulin-5, in prolapsed human vaginal tissue that correlates with POP severity [23, 26, 30]. In our study, elastin mRNA and protein expression in the vagina in cell-injection compared to saline-injection group was inconsistent. These findings suggest that the improvement in vaginal function after pSMCs injection may not be directly associated with changes in elastin. In contrast, urethral tissues demonstrated significantly increased collagen I and elastin expressions in cell-injection group compared to saline-injection groups. These observations of the urethral tissues from the current study are consistent with our previous study where we observed the same effect of pSMCs on the urethra of the SUI rat model [8]. These data either suggest a differential response to pSMCs between the urethra and the vagina, or that, pSMC dose may need further optimization to achieve significant effects in the vagina.

### Limitation of our study:

1. Limited number of patient pSMC lines tested. The lack of consistently significant differences is likely due to patient-to-patient variability. We selected two patients in their early 40’s and one in her early 70’s of age to examine if age may affect the results. We did not see a pattern to suggest this effect. The lack of evidence that cells from the oldest participant performed poorly is consistent with data confirming that the iPSC reprogramming process may rejuvenate cells[31, 32]. Future larger sample size is recommended to evaluate the effect of donor age on pSMC *in vivo* behavior.
2. We observed that the injection of pSMC altered ECM in the urethra significantly, while the results in the vagina were less consistent. In the BLI study, cells could not be detected after week 1. Taken together, we suspect that the cell dose of 2×10^6^ may be insufficient for the vagina. The injected cells are more likely to migrate to the surrounding areas further decreasing the effect on the vagina. Future studies should consider increasing cell numbers and methods that localize the cells to the vagina.
3. Due to the small size of the rat vagina, we needed to use different portions for organ bath myography, PCR, and protein testing. We performed a pilot study (not described in this manuscript) to evaluate the effect of organ bath myography on tissue mRNA expression. Because this pilot study found that organ bath affected RNA expression, we used the proximal vagina for PCR and histology, and the middle vagina for organ bath and protein evaluation. Despite this, using different parts for the vagina may have introduced variability which affected our comparisons.
4. We used h-CALD staining to examine smooth muscle thickness to investigate whether there is an association between the function of the vagina and smooth muscle content after the injection of pSMC. This is a qualitative, 2D assessment with high variability. This study was limited by vaginal tissue size, future studies would benefit from more quantitative methods for measuring smooth muscle content such as volumetric density of smooth muscle[16, 33, 34].
5. We were not able to randomize assignment of the rats to the different experimental groups due to Pandemic related rat supply. While the qualitative elastin scores were done with the evaluators blinded to the experimental groups, all other assays were performed without blinding. This was done to prevent errors of assignments as there were many different assays being performed simultaneously. These may have introduced confounders to the results.

## Conclusions

In this study, we observed that human iPSC-derived pSMCs improved contractility of the vagina in a vaginal-injury rat model. Additionally, there was an increase in collagen I protein expression in the vagina suggesting a improvement in vaginal tensile strength. These observations support further efforts in translating hiPSC-derived pSMCs into a potential therapeutic option for regenerating the surgically-injured vagina in women who suffer recurrent prolapse after surgery.

## Figures and Tables

**Table 1.** Primers used for *Alu*-seq-PCR and RT-qPCR

	Forwoard primer	Reverse Primer
human-specific DNA sequence *Alu*	GGTTCAAGCGATTCTCCTGC	GGTGAAACCCCGTCTCTACT
*elastin*	ATCGGTGGCTTAGGAGTCTCAACA	TGGAAGACCGACACCAGGAACTTT
*collagen I*	GAAGGCAACAGTCGATTCACC	GACTGTCTTGCCCCAAGTTCC
*collagen III*	TGATGGGATCCAATGAGGGAGA	GAGTCTCATGGCCTTGCGTGTTT
*GAPDH*	GCCAGCCTCTCTCATAGACA	TGGTAACCAGGCCGTCCGATA

**Table 2.** Gene (RT-qPCR) and protein (ELISA) expression of collagen I, collagen III, col I/III, and elastin of vagina in different pSMCs groups compared to VSHAM group.

RT-qPCR of Vagina Proximal portion			
Marker	A pSMC cells vs. VSHAM	B pSMC cells vs. VSHAM	C pSMC cells vs. VSHAM
Collagen I	0.011±0.036 vs. 0.462±0.311 P=0.0001↓*	0.317±0.355 vs. 0.180±0.127 P=0.5478↑	0.594±0.450 vs. 0.366±0.272 P=0.3123↑
Collagen III	0.042±0.107 vs. 0.877±0.780 P=0.0001↓*	1.242±1.303 vs. 1.146±1.045 P=0.8376↑	0.729±0.530 vs. 0.858±0.736 P=0.7928↓
Col I/III	0.107±0.154 vs. 0.620±0.308 P=0.0002↓*	0.456±0.435 vs. 0.232±0.137 P=0. 0825↑	0.742±0.343 vs. 0.415±0.232 P=0.0194↑*
Elastin	0.050±0.063 vs 0.464±0.458 P=0.0005↓*	0.039±0.037 vs. 0.019±0.017 P=0.1418↑	0.328±0.225 vs. 0.447±0.299 P=0.2122↓
ELISA of Vagina Middle portion			
Marker	A pSMC cells vs. VSHAM	B pSMC cells vs. VSHAM	C pSMC cells vs. VSHAM
Collagen I	2.171±0.873 vs. 1.283±0.433 P=0.0289↑*	1.113±0.258 vs. 0.694±0.138 P=0.0033↑*	0.873±0.294 vs. 0.694±0.138 P=0.1740↑
Collagen III	93.91±69.16 vs. 126.4±80.18 P=0.2815↓	46.95±21.77 vs. 48.21±24.11 P=0.8883↓	66.59±17.69 vs. 48.21±24.11 P=0.1223↑
Col I/III	0.073±0.103 vs. 0.033±0.048 P=0.1029↑	0.035±0.035 vs. 0.021±0.014 P=0.2729↑	0.014±0.006 vs. 0.021±0.014 P=0.2364↓
Elastin	159.2±59.95 vs. 170.4±36.50 P=0.5181↓	48.23±19.71 vs. 60.57±16.64 P=0.2048↓	65.75±15.96 vs. 60.57±16.64 P=0.4281↑

*: P=0.05

**Table 3.** Gene (RT-qPCR) and protein (ELISA) expression of collagen I, collagen III, col I/III, and elastin of the urethra in different pSMCs groups compared to VSHAM group.

RT-qPCR of Urethra Middle portion			
Marker	A pSMC cells vs. VSHAM	B pSMC cells vs. VSHAM	C pSMC cells vs. VSHAM
Collagen I	10.87±3.599 vs. 0.553±0.450 P=0.0001↑*	15.55±7.151 vs. 0.816±0.171 P=0.0001↑*	26.16±14.12 vs. 6.669±9.110 P=0.0008↑*
Collagen III	955.3±375.0 vs. 0.367±.0374 P=0.0001↑*	506.3±188.6 vs. 0.448±0.753 P=0.0001↑*	1944.3±968.2 vs.333.9±621.4 P=0.0008↑*
Col I/III	0.0122±0.0034 vs.2.926±3.034 P=0.0006↓*	0.029±0.006 vs. 8.995±13.96 P=0.0022↓*	0.012±0.002 vs. 1.430±1.517 P=0.0036↓*
Elastin	8.586±5.258 vs. 0.018±0.039 P=0.0001↑*	5.262±5.034 vs. 0.099±0.285 P=0.0001↑*	53.41±29.44 vs. 9.248±12.38 P=0.0005↑*
ELISA of Urethra Distal portion			
Marker	A pSMC cells vs. VSHAM	B pSMC cells vs. VSHAM	C pSMC cells vs. VSHAM
Collagen I	2.530±0.684 vs. 2.021±0.433 P=0.0477↑*	0.957±0.240 vs. 0.879±0.302 P=0.4233↑	1.038±0.350 vs. 0.888±0.422 P=0.2210↑
Collagen III	15.54±5.805 vs. 11.92±4.182 P=0.0120↑*	1.786±1.371 vs. 3.052±1.902 P=0.0684↓	5.825±2.766 vs. 6.402±3.133 P=0.5316↓
Col I/III	0.062±0.014 vs. 0.081±0.017 P=0.0043↓*	0.832±0.610 vs. 0.419±0.333 P=0.0348↑*	0.210±0.062 vs. 0.178±0.093 P=0.1086↑
Elastin	42.53±37.17 vs. 88.37±30.61 P=0.0017↓*	41.96±22.84 vs. 36.80±21.88 P=0.7237↑	94.16±44.35 vs. 53.44±31.22 P=0.0114↑*

*: P=0.05

## Data Availability

Data are available on request. Numerical data on organ bath myography are provided in Supplemental Table 2.

## Abbreviations

iPSCs: Pluripotent stem cells
pSMCs: Progenitors of smooth muscle cells
POP: Pelvic organ prolapse
SUI: Stress urinary incontinence
MSCs: Mesenchymal stem cells
ECM: extracellular matrix
FBS: Fetal bovine serum
RNU: Rowett Nude rats
VSHAM: The group of vaginal-injury model injected with saline
BLI: Bioluminescence imaging
RT-qPCR: Reverse-Transcription Quantitative Polymerase Chain Reaction
ELISA: Enzyme-linked immunosorbent assay
OCT: Optimal cutting temperature
h-CALD: h-Caldesmon
Col I/III: Ratio of collagen I/III
SMDCs: Skeletal muscle-derived cells
